# Motile mosquito stage malaria parasites: ready for their close‐up

**DOI:** 10.15252/emmm.202113975

**Published:** 2021-03-31

**Authors:** Ashley Vaughan

**Affiliations:** ^1^ Center for Global Infectious Disease Research Seattle Children's Research Institute Seattle WA USA

**Keywords:** Microbiology, Virology & Host Pathogen Interaction

## Abstract

Many stages of the complex Plasmodium parasite life cycle, the eukaryotic pathogen that causes malaria, are extracellular and motile. This motility is essential for life cycle progression, and two studies in this issue of *EMBO Molecular Medicine* (Hopp *et al*, 2021; Ripp *et al*, 2021) examine the motility of two of these life cycle stages. These are the ookinete, which develops in the midgut of an infected mosquito vector, and the sporozoite, which is injected into the skin of an unsuspecting host by an infected mosquito, initiating the parasite life cycle in the human. Therapeutic targeting of the ookinete and sporozoite (Duffy & Patrick Gorres, 2020), which are profound bottlenecks in the life cycle, has recently received a great deal of attention in our battle to prevent the 400,000 deaths from malaria that occur every year (WHO, 2020).

## Graphical abstract

A. Vaughan discusses malaria parasite ookinete and sporozoite motility and two models to pre‐clinically test malaria intervention strategies as reported by Ripp *et al* (2021) and Hopp *et al* (2021), in this issue of *EMBO Mol Med*.

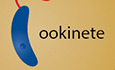

The extremely complex malaria life cycle alternates between humans and mosquitoes. Immature gametes known as gametocytes are ingested in a human malaria infectious blood meal by the mosquito vector. The blood meal is essential for mosquito egg production and used by the parasite as a route of transmission. Within the blood meal bolus, the gametocytes mature into haploid gametes, and gamete fusion results in the formation of a diploid zygote, which then differentiates into a tetraploid ookinete. The motile ookinete moves through, and exits the blood meal bolus, comes into contact with the mosquito midgut, traverses the midgut wall, and attaches to the basal lamina. The ookinete now transforms into an oocyst which matures and releases thousands of sporozoites, which are then carried in the mosquito hemolymph to the salivary gland (Fig 1A). The sporozoite traverses into the lumen of the salivary gland and awaits the acquisition of the next blood meal by the mosquito. During the blood meal, salivation introduces anticoagulants and the sporozoites into the dermis of the bite site. Sporozoite deposition into the human host leads to its rapid motility and dispersal from the bite site and eventually to blood vessel entry. Once in the bloodstream, the sporozoite is carried throughout the body and exits within the liver sinusoid and ultimately infects a single hepatocyte where it develops, undergoes enormous replication, ultimately releasing tens of thousands of merozoites that invade red blood cells. This initiates the blood stage of the life cycle, responsible for all morbidity and mortality associated with the disease.

Very few ookinetes successfully develop into oocysts, and very few sporozoites successfully infect the liver, and thus, these life cycle stages are ideal targets for intervention. Indeed, antibodies that recognize the ookinete surface and the sporozoite surface can prevent their motility and block life cycle progression. To further understand how the ookinete and sporozoite reach their destinations, the studies by Ripp *et al* (2021) and Hopp *et al* (2021) used complementary *in vivo* and *in vitro* techniques.

**Figure 1:**
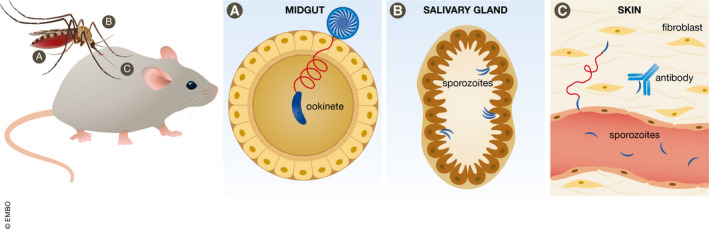
Plasmodium ookinetes and sporozoites show unique motility patterns (A) After an infectious blood meal, Plasmodium gametes mature and fuse to form a zygote which develops into a motile ookinete. The ookinete slowly makes its way through the blood meal, crosses the midgut epithelium, and develops into an oocyst. The oocyst releases mature sporozoites into the hemolymph, and they travel to the salivary gland. (B) Salivary gland sporozoites lie in wait and are injected into the skin of the vertebrate host during a further infectious blood meal. (C) Within the dermis of the skin, highly motile salivary gland sporozoites move through the dermis and, if successful, enter a skin capillary through the endothelial cell layer and travel in the bloodstream to the liver to continue the life cycle. Antibodies recognizing the sporozoite can limit its movement and entry into the vasculature and therefore prevent ongoing life cycle progression and are effective vaccine targets.

To study the ookinete and sporozoite, Ripp *et al* (2021) deferred to the rodent malaria parasite *Plasmodium berghei*, an excellent model of the human malaria parasite, and introduce tunable polyacrylamide gels that allow measurements of motility on both two‐ and three‐dimensional substrates. As opposed to Matrigel and collagen, which are often used as substrates for motility measurements, these polyacrylamide hydrogels can be made with a defined elasticity and pore size by adjusting monomer and crosslinker concentration. Increasing the acrylamide concentration increases the stiffness of the matrix and increasing the bisacrylamide concentration leads to smaller pores. This then allowed for the mimicking of tissues and cell types. Ookinetes were visualized on non‐coated planar polyacrylamide hydrogel sandwiches to establish a confined environment. Excitingly, ookinetes meandered in a circular fashion for over 24 h on very soft substrates, which mimic the blood meal bolus within which they develop, suggesting that the sandwich was indeed mimicking the *in vivo* setting. An increase in substrate stiffness led to a change in the migration path, to a more linear path, suggestive of the ookinetes journey across the stiffer midgut epithelium, and final resting place at the midgut basal lamina.

Unlike the ookinete which maintains motility until it transforms into an oocyst, the motile sporozoite is presumably motionless, biding its time in the salivary gland, lying in wait for its mosquito vector to take a blood meal (Fig 1B). Only then, within the skin dermis, does the sporozoite immediately gain motility and seeks out a blood vessel for entry and travel to the liver. Sporozoites are fast, achieving speeds of approximately 1 μm/s. Traditionally, sporozoite motility has been studied *in vitro* in a two‐dimensional setting. Simply placing sporozoites in media on a bovine serum albumin‐coated coverslip at 37°C leads to attachment and rapid helical motility, resulting in multiple circular trails over an hour. Ripp *et al* (2021) sandwiched infected salivary glands between a soft polyacrylamide hydrogel and glass coverslip, and observed sporozoites moving into the hydrogel, where they then moved in helical trajectories. With decreasing pore size, sporozoites slowed down and became immotile and eventually failed to enter the hydrogels. Interestingly, bovine serum albumin was essential for sporozoites to move inside the hydrogels, but not necessary for sporozoites to enter hydrogels, suggesting that substrate is sufficient for surface motility. The studies show that three‐dimensional polyacrylamide hydrogels with large pore sizes and increased stiffness can be used to mimic sporozoite migration in the skin. This *in vitro* system could thus be used for the testing of drugs and antibodies that impede sporozoite motility and prevent the sporozoite leaving the skin.

Once they locate a blood vessel, sporozoites must be carried to the liver sinusoid and enter the liver parenchyma. How do sporozoites “turn‐off” their motility in order to cross the sinusoid? Ripp *et al* (2021) hypothesized that sporozoites were highly sensitive to substrate elasticity possibly to avoid adhesion to soft endothelial cells. Indeed, sporozoites showed no motility on cultured endothelial cells, although they were motile on stiffer fibroblasts. Thus, sporozoites are less capable of movement on endothelial cells, allowing sporozoites to enter and exit the vasculature to gain entry to the hepatocyte, its final resting place (Fig 1C).

The elegant study by Hopp *et al* (2021) utilized the human malaria *P. falciparum* sporozoite and a mouse model of skin to initially probe the *in vivo* nature of the injected sporozoite at the bite site. Fascinatingly, the skin did not provide a species‐specific barrier for blood vessel entry, since *P. falciparum* sporozoites entered blood vessels as effectively as their rodent malaria counterparts, *P. yoelii* and *P. berghei*. Of importance, the passive transfer of antibodies into recipient mice demonstrated that targeting the *P. falciparum* circumsporozoite protein (Fig 1C), which covers the surface of the sporozoite, prevented their entry into blood vessels, and thus, this model can be used for the preclinical testing of antibodies that prevent sporozoite access to the liver.

Using exquisite *in vivo* imaging techniques (Hopp, Chiou *et al,* 2015) and transgenic fluorescent sporozoites, Hopp *et al* (2021) were able to accurately measure sporozoite motility. This ranged from 1 to 3 μm/s for up to an hour or longer, demonstrating that the dermal inoculation site is where the extracellular parasite spends the largest amount of time in the mammalian host. Motility was initially linear but became more confined to allow for contact with blood vessels. During the two hours of sporozoite visualization, there was a gradual decrease in displacement with a median displacement of 20 µm at 5 min after injection with highest final displacements seen at 10 min. Amazingly, maximal displacements between 5 and 20 min ranged between 75 and 200 μm. Over time, sporozoite motility did decline although only by about two‐fold over the 2‐h period. Of note, *P. yoelii* sporozoites maintained motility for longer than their *P. berghei* counterparts, and in the mouse model used, *P. yoelii* gives rise to higher infection rates to the liver after mosquito bite infections. The authors suggest that this could be due to the prolonged period the sporozoite actively spends in the dermis, giving it longer to enter the blood stream and enter the liver.

*P*. *falciparum* sporozoites were clearly shown to circle around CD31^+^ blood vessels, presumably to aid their entry into the vessel and 5.5% of the injected sporozoites entered blood vessels during the time of imaging: four‐minute videos recorded at five‐ and 20‐min post‐inoculation. A further 1.7% of sporozoites entered the lymphatics. To further mimic the human host, grafted human skin was used in mouse experiments, resulting in the retention of the human vasculature and the restoration of the blood supply to the graft through spontaneous anastomosis of murine and human microvessels. Confirmation of the presence of human blood vessels was achieved with antibodies to human CD31. The xenografts led to a lower overall displacement over the first 5–10 min after inoculation of sporozoites. Hopp *et al* (2021) hypothesized that in the xenografts, the anastomoses could result in larger, more tortuous vessels, such that sporozoites in the grafted skin could be in closer proximity to vessels and displaying the more constrained motility observed when *P. berghei* sporozoites were near vessels (Hopp *et al,* 2015). This would also agree with the observations of Ripp *et al* (2021) who showed significantly decreased motility on endothelial cells. The authors thus measured track straightness, the ratio of displacement to track length, to quantify the confinement of motility in xenografts compared to mouse skin. Indeed, *P. falciparum* sporozoite track straightness in the xenograft was significantly reduced, showing that *P. falciparum* sporozoites move in more constrained circular paths in the grafted human skin, due to the tortuous vessel formation in the xenograft. This was confirmed in studies that showed that sporozoites were less constrained in the *ex vivo* skin grafts used for transplantation. The authors importantly showed that *P. falciparum* sporozoites invade blood and lymphatic vessels in mouse skin at a rate comparable to the rodent parasites and were seen to interact with blood vessels in a similar fashion. This suggests that sporozoite recognition of blood vessels is likely based on molecules that are shared among different host species although the receptor(s) is/are unknown. Thus, this model can be used to cheaply study sporozoite interventions to human malaria sporozoite exit from the skin and would provide excellent preclinical data to support further experimentation in humans.

These two complementary papers provide significant insight into malaria parasite ookinete and sporozoite motility. The two models can be cheaply and easily used by interested parties in the malaria community to test novel interventions to this still devastating and lethal disease.
